# Prenatal hypoxia leads to hypertension, renal renin-angiotensin system activation and exacerbates salt-induced pathology in a sex-specific manner

**DOI:** 10.1038/s41598-017-08365-4

**Published:** 2017-08-15

**Authors:** S. L. Walton, H. Bielefeldt-Ohmann, R. R. Singh, J. Li, T. M. Paravicini, M. H. Little, K. M. Moritz

**Affiliations:** 1 School of Biomedical Sciences, The University of Queensland, St Lucia, Brisbane, Queensland Australia; 2 School of Veterinary Science, The University of Queensland, Gatton, Queensland Australia; 3 Department of Physiology, Monash University, Clayton, Victoria, Australia; 4 Institute for Molecular Bioscience, The University of Queensland, St Lucia, Brisbane, Queensland Australia; 5 School of Health and Biomedical Sciences, RMIT University, Melbourne, Victoria, Australia; 6 Murdoch Childrens Research Institute, Parkville, Victoria, Australia; 7 Child Health Research Centre, The University of Queensland, Brisbane, Australia

## Abstract

Prenatal hypoxia is associated with growth restriction and adverse cardiovascular outcomes. Here, we describe renal and cardiovascular outcomes in ageing mouse offspring prenatally exposed to hypoxia (12% O_2_) from embryonic day 14.5 until birth. At 12 months of age, both male and female offspring exposed to prenatal hypoxia had elevated mean arterial pressure. Glomerular number was reduced by 25% in hypoxia-exposed male, but not female, offspring and this was associated with increased urinary albumin excretion, glomerular hypertrophy and renal fibrosis. Hypoxia-exposed offspring of both sexes were more susceptible to salt-induced cardiac fibrosis, however, renal fibrosis was exacerbated by high salt in males only. In male but not female hypoxia-exposed offspring, renal *renin* mRNA was increased at weaning. By 12 months, renal *renin* mRNA expression and concentrations were elevated in both sexes. mRNA expression of *At*_*1a*_ *R* was also elevated in male hypoxia-exposed offspring at 12 months. These results demonstrate that prenatal hypoxia programs elevated blood pressure and exacerbates salt-induced cardiovascular and renal pathology in a sex specific manner. Given sex differences observed in RAS expression and nephron number, future studies may consider RAS blockade as a therapeutic target in this model.

## Introduction

*In utero* insults, such as reduced oxygen and nutrient supply to the fetus, lead to growth restriction and predisposition to cardiovascular, renal and metabolic diseases in later life^1^. Growth restriction is frequently associated with impaired organ development^2^, meaning offspring may be born with organs lacking the robustness required for adequate support throughout the lifespan. Fetal hypoxia is a common pregnancy complication that arises from a wide range of circumstances including, but not limited to, placental insufficiency, high altitude living and maternal factors such as smoking and pulmonary disease^3, 4^. In the chronically hypoxic fetus, blood flow is shunted towards the brain, heart and adrenal glands at the expense of peripheral organs such as the kidneys^5^; however, the long-term renal outcomes for these offspring are unknown.

We have previously reported that both male and female mouse offspring exposed to prenatal hypoxia (12% O_2_, reduced from 21% O_2_) in late gestation are growth-restricted with impaired placental vascularisation^6, 7^. Offspring develop endothelial dysfunction by 12 months of age^8^, which in the human population is associated with increased risk of cardiovascular events^9^. Studies in rat offspring from pregnancies complicated by hypoxia have also identified ventricular and aortic wall thickening^10^, susceptibility to cardiac ischemia-reperfusion injury^11^ and peripheral vascular dysfunction^12, 13^. Less focus has been placed on renal outcomes following fetal hypoxia, which is surprising given the kidney is highly susceptible to *in utero* insults and is critical for long-term maintenance of blood pressure^2, 14^.

A congenital nephron deficit, a common outcome following an *in utero* perturbation, reduces the filtration surface area of the kidney, which in the human population is associated with risk of hypertension and progressive kidney damage^15^. Reduced nephron number is frequently reported to co-exist with hypertension in a range of animal models of suboptimal fetal growth including uteroplacental insufficiency^16^, maternal alcohol consumption^17^, and malnutrition^18^. Alterations to the renal renin angiotensin system (RAS) are major contributors to impaired renal development and subsequent onset of hypertension in offspring^19, 20^. It has been recently suggested that activation of the RAS occurs in a temporal and sex-specific manner, with earlier activation in males. Notably, males more are more frequently and/or severely affected compared to females^19^. RAS blockade is currently the first-line therapy for treating hypertension in humans, and attenuates age-related elevations in blood pressure. Furthermore, RAS blockade in growth restricted rats abolishes elevated blood pressure^21^, which supports a role of the RAS in programmed hypertension.

Offspring born from compromised pregnancies frequently display increased susceptibility to disease risk factors^22^, with ageing or a poor diet often unmasking or exacerbating disease outcomes. The postnatal ‘second-hit’ of a diet high in salt has been shown to exacerbate disease outcomes following maternal protein restriction^18^ and a congenital nephron deficit^23^ in rodents. Indeed, in our model, offspring exposed to prenatal hypoxia and a postnatal high salt diet had marked stiffening of the resistance vasculature and altered extracellular matrix composition in the aorta^8^, consistent with cardiovascular disease. However, no study has yet investigated whether cardiac and renal pathologies following prenatal hypoxia are exacerbated by a high salt diet and ageing.

The aim of this study was to utilise our mouse model of hypoxia during late gestation^6–8^ to assess blood pressure, renal function and cardiac/renal pathology in male and female offspring. We also examined if these outcomes were associated with sex-specific alterations in the renal RAS in young and aged animals. Given the postnatal environment may unmask or exacerbate disease outcomes, we also hypothesised that hypoxia-exposed offspring would have increased susceptibility to tissue damage due to high dietary salt intake.

## Results

### Body/organ weights from E18.5 to 2 months of age

As reported previously^8^, body weight was reduced by hypoxia exposure at E18.5 and P21 (Table 1)^6, 8^. Kidney weight was reduced in hypoxia-exposed offspring at E18.5 (control male: 10.69 ± 0.61 mg, control female: 10.02 ± 0.54 mg, hypoxia male: 8.96 ± 0.50 mg, hypoxia female: 8.77 ± 0.99 mg; P_treatment_ = 0.04); and P21 (Table 1) compared to controls; kidney weight corrected for body weight was similar between treatment groups. Heart weight, absolute or corrected for body weight, did not differ between treatment groups at E18.5 (control male: 7.78 ± 0.42 mg, hypoxia male: 7.75 ± 0.47 mg, control female: 7.90 ± 0.31 mg, hypoxia female: 6.81 ± 0.41 mg; P_treatment_ = 0.2) or P21 (Table 1). By 2 months of age, hypoxia-exposed offspring had the same body weight as controls (control male: 35.5 ± 0.8 g, hypoxia male: 34.9 ± 0.6 g, control female: 27.6 ± 0.7 g, hypoxia female: 28.2 ± 0.7 g; P_treatment_ = 0.98).

**Table 1:** Offspring body/organ weights, renal morphology and renal RAS *mRNA* expression at postnatal day 21.

	Control		Hypoxia		Two-way ANOVA		
	*Male*	*Female*	*Male*	*Female*	*Treatment*	*Sex*	*Interaction*
**Body and organ weights**							
*Bw* (*g*)	11.86 ± 0.3	11.20 ± 0.3	10.95 ± 0.3	10.39 ± 0.4	P = 0.01	n.s.	n.s.
*Brain* (*mg*)	361 ± 9	360 ± 10	357 ± 17	379 ± 9	n.s.	n.s.	n.s.
*Brain*:*bw*	30.7 ± 1.4	32.5 ± 1.1	32.4 ± 1.8	37.7 ± 2.2	P = 0.04	P = 0.03	n.s.
*Heart* (*mg*)	75.2 ± 2.8	72.2 ± 1.6	72.4 ± 2.7	70.8 ± 3.3	n.s.	n.s.	n.s.
*Heart*:*bw*	6.27 ± 0.1	6.42 ± 0.1	6.5 ± 0.2	6.6 ± 0.1	n.s.	n.s.	n.s.
*Kidney* (*mg*)	83.1 ± 2.8	78.6 ± 2.8	75.9 ± 2.2	73.2 ± 3.4	0.03	n.s.	n.s.
*Kidney*:*bw*	6.94 ± 0.2	6.96 ± 0.1	6.81 ± 0.1	6.86 ± 0.1	n.s.	n.s.	n.s.
**Renal morphology**							
*Glomerular number*	12888 ± 515	9854 ± 432	9782 ± 517**	9753 ± 440	P = 0.002	P = 0.004	P = 0.004
*Glomerular area* (*um* ^2^)	1964 ± 130	2140 ± 185	1797 ± 72	1778 ± 128	n.s.	n.s.	n.s.
**Renal RAS** ***mRNA*** **expression**							
*Renin*	1.22 ± 0.27	1.45 ± 0.25	2.17 ± 0.43*	1.14 ± 0.17	n.s.	n.s.	P = 0.03
*At*_*1a*_ *R*	1.02 ± 0.07	1.13 ± 0.09	1.16 ± 0.11	1.02 ± 0.09	n.s.	n.s.	n.s.
*Ace*	1.08 ± 0.16	1.16 ± 0.19	1.37 ± 0.17	0.88 ± 0.08	n.s.	n.s.	n.s.

Values are mean ± SEM. Organ/body weight (bw): N = 2 offspring from 11 litters/group. Glomerular number/area: N = 8–9/group. *mRNA* expression: N = 9–12/sex/litter. Effect of treatment, sex or interaction (treatment x sex) was evaluated by two-way ANOVA. *P < 0.05, **P < 0.001 comparing male control/hypoxia offspring with a Sidak *post hoc*. Not significant (n.s.).

### Glomerular number, electrolyte excretion and renal RAS expression in young mice

Glomerular number was significantly affected by both prenatal hypoxia and sex (Table 1). *Post hoc* analysis demonstrated males had more glomeruli than females, but hypoxia-exposed males had 25% less glomeruli compared to control males (Table 1). Glomerular area was equivalent between treatment groups at P21 (Table 1). Urine output and electrolyte excretion did not differ between treatment groups at 2 months of age (see Supplementary Fig. S1). *Renin* mRNA expression was increased in kidneys of male hypoxia-exposed offspring at P21 compared to male controls (Table 1; P < 0.05 from Sidak *post hoc*). No differences in *At*_*1a*_ *R* or *Ace* mRNA expression in kidneys of P21 offspring were observed between groups (Table 1). Albumin excretion did not differ between control and hypoxia-exposed mice at 4 months of age (control male: 8.97 ± 1.5 μg/24 h, hypoxia male: 8.2 ± 1.5 μg/24 h, control female: 8.09 ± 1.9 μg/24 h, hypoxia female: 6.6 ± 1.4 μg/24 h).

### Blood pressure measurements

**Figure 1:**
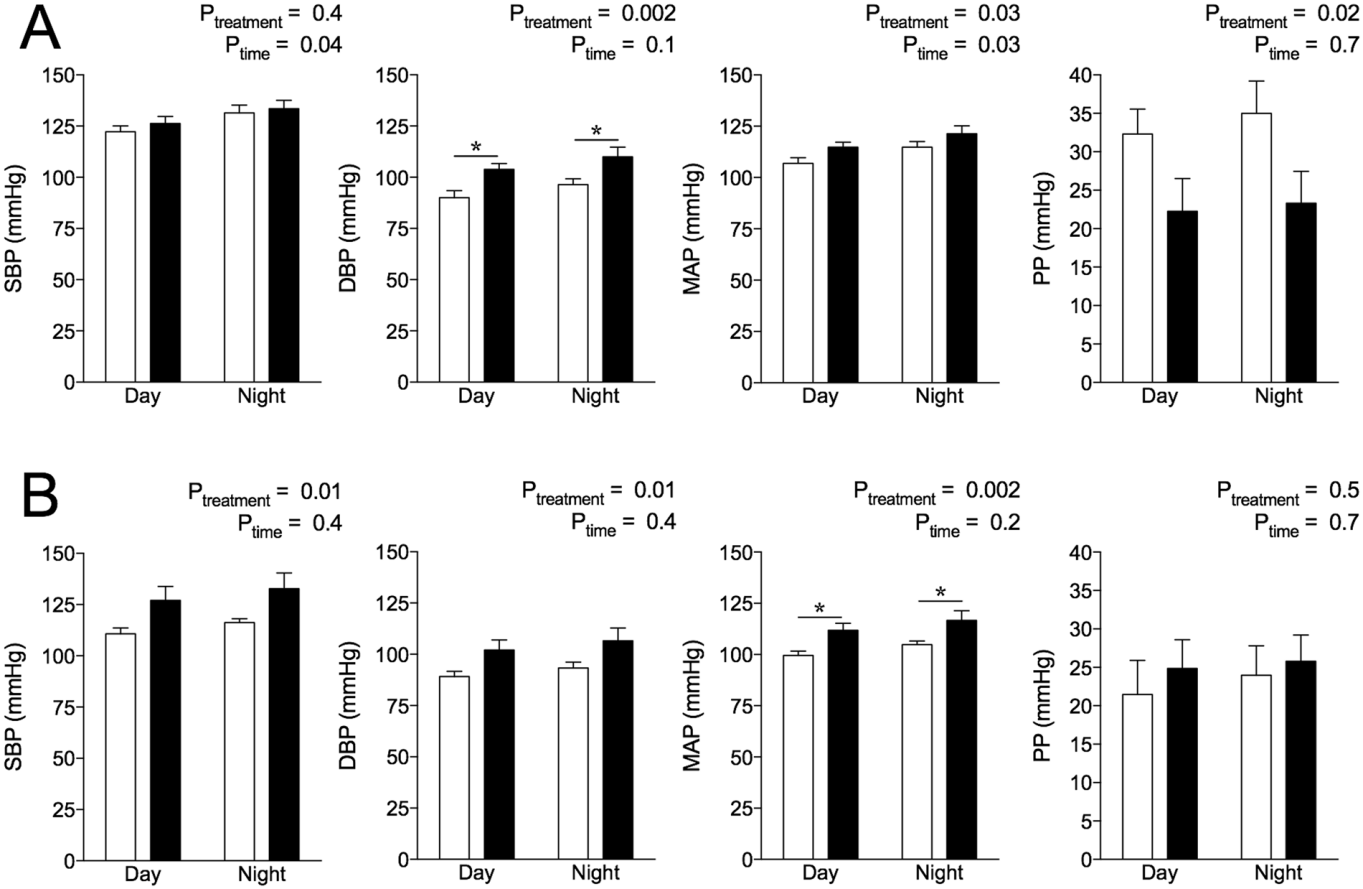
Blood pressure (mmHg) profiles of offspring at 12 months of age. Systolic blood pressure (SBP), diastolic blood pressure (DBP), mean arterial pressure (MAP) and pulse pressure (PP) in male (**A**) and female (**B**) offspring. Data analysed via two-way ANOVA. Values are mean ± SEM. *P < 0.05 by Sidak *post hoc*. Control: open bars; hypoxia: closed bars.

Mean arterial pressure (MAP) was elevated in male (Fig. 1A) and female (Fig. 1B) hypoxia-exposed offspring fed the normal salt (NS) diet with respect to controls. In hypoxia-exposed males, increase in MAP was reflected by an increase in diastolic blood pressure (DBP) with no change in systolic blood pressure (SBP), leading to reduced pulse pressure (PP) (Fig. 1A). Female hypoxia-exposed offspring had elevated SBP and DBP, with no difference in PP (Fig. 1B). Heart rate (HR) was not affected by hypoxia (see Supplementary Fig. S2A,B). The MANOVA analysis (see Supplementary Table S1) showed significant effects of sex, with females having decreased SBP and MAP compared to males. HR was elevated in females compared to males (see Supplementary Fig. S2A,B). SBP, DBP, MAP and activity were all significantly affected by time, as expected with circadian variations (see Supplementary Table S1). Activity in male control offspring and all female offspring groups increased during the night periods (see Supplementary Fig. S2A,B). A significant treatment-period interaction in activity was observed, as activity in male hypoxia-exposed offspring was blunted during the night period compared to control males.

Restraint stress increased MAP above baseline levels in male and female offspring (see Supplementary Fig. S3A,B) in both treatment groups. No differences in ∆HR, PP, SBP or DBP were observed between treatment groups during restraint stress in either sex (see Supplementary Fig. S3A,B). Males compared to females, irrespective of prenatal treatment, had an increased ∆ HR (P_sex_ = 0.02) and ∆ MAP (P_sex_ = 0.049) during restraint. There was a tendency for increased ∆ SBP (P_sex_ = 0.06) and ∆ DBP (P_sex_ = 0.07) in males compared to females during restraint, although this did not reach significance.

### Intrarenal renin-angiotensin system measurements at 12 months of age

**Figure 2:**
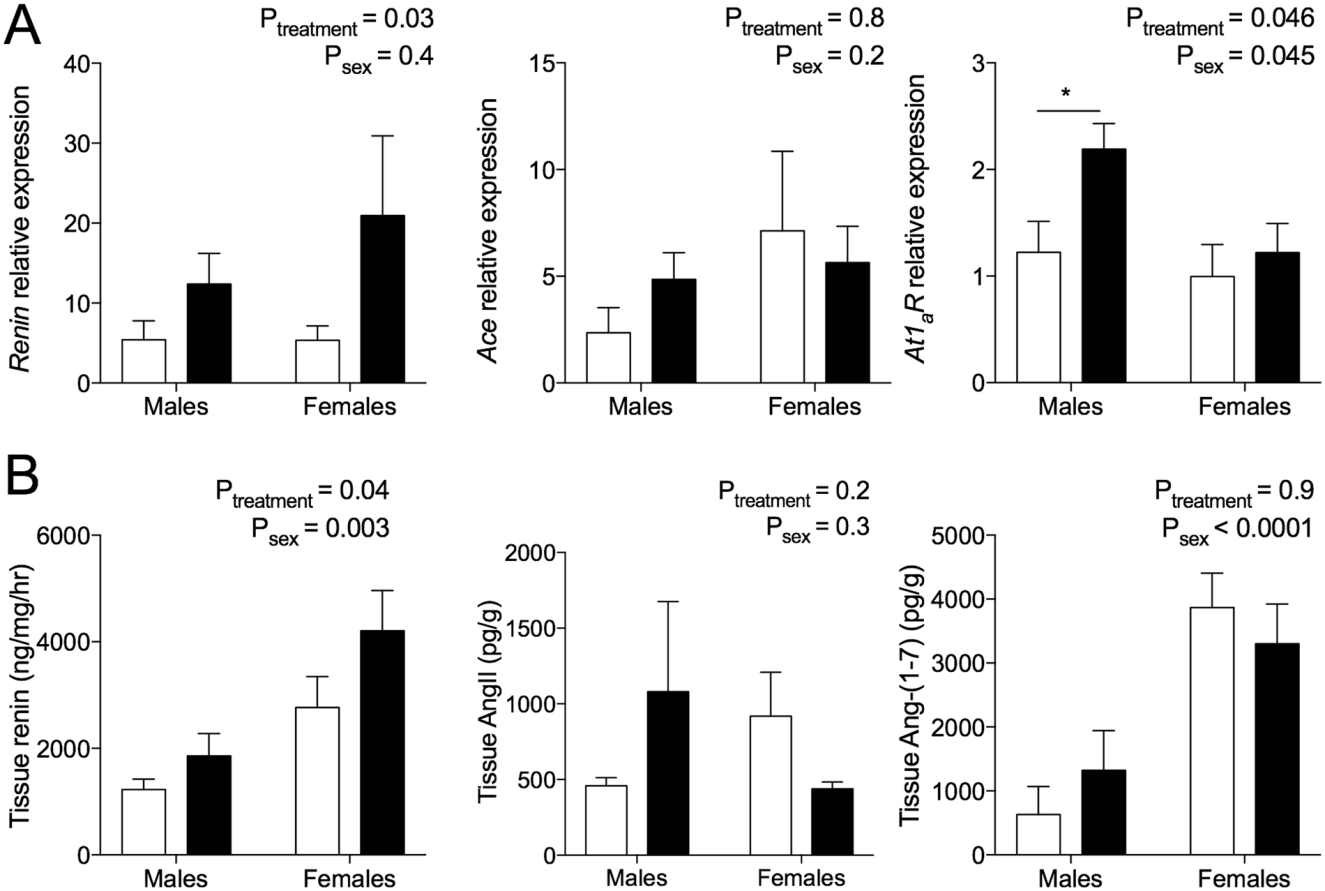
Renal renin-angiotensin system expression at 12 months of age. (**A**) *Renin*, *At*_*1a*_ *R* and *Ace* relative mRNA expression presented relative to the control male, with ß-actin as a housekeeper. Male: 7–11/group; female: N = 5–6/group. (**B**) Renal tissue concentrations of renin, angiotensin II and angiotensin-(1–7). Male: N = 11/group; female: N = 7–9/group. Data analysed via two-way ANOVA. Values are mean ± SEM. *P < 0.05 by Sidak *post hoc*. Control: open bars; hypoxia: closed bars.

*Renin* mRNA expression was increased in hypoxia-exposed male and female offspring compared to controls (Fig. 2A). *At*_*1a*_ *R* mRNA expression was elevated in male hypoxia-exposed offspring compared to male controls, but was not different between female treatment groups (Fig. 2A; P < 0.05 from Sidak *post hoc*). *Ace* mRNA expression did not differ between groups (Fig. 2A). Renal renin concentrations were greater in hypoxia-exposed offspring of both sexes compared with controls (Fig. 2B). Renal renin concentration was higher in females than males in all groups. Renal angiotensin II content was not different between treatment groups or sexes, while renal angiotensin-(1–7) content was greater in females compared to males, irrespective of treatment group (Fig. 2B).

### Urine and plasma analysis at 12 months of age

Urinary excretion of sodium and chloride did not differ between prenatal treatment groups but was increased by the high salt (HS) diet (Table 2). *Post hoc* analysis revealed hypoxia-exposed male offspring fed HS excreted more chloride than control counterparts (Table 2). Potassium excretion did not differ between prenatal treatment and dietary groups (Table 2). Albumin excretion in male hypoxia-exposed offspring was elevated ~130% compared to control counterparts irrespective of postnatal diet (Table 2). No differences in albumin excretion were observed in female offspring at 12 months of age. Plasma cystatin C levels were not affected by prenatal treatment, but were reduced in male offspring fed the HS diet and trended towards a decrease in female offspring fed the HS diet (Table 2).

**Table 2:** Offspring body and organ weights and renal parameters at 12 months of age.

	Normal Salt		High Salt		Two-way ANOVA		
	*Control*	*Hypoxia*	*Control*	*Hypoxia*	*Treatment*	*Diet*	*Inter*-*action*
**MALES**							
***Body and organ weights***							
Bw (g)	55 ± 2	55 ± 2	58 ± 3	57 ± 2	n.s.	n.s.	n.s.
Kidney (mg)	724 ± 35	715 ± 19	841 ± 75	912 ± 26	n.s.	P = 0.002	n.s.
Kidney:bw	13.2 ± 0.6	12.7 ± 0.4	14.6 ± 1.3	16.2 ± 0.7	n.s.	P = 0.02	n.s.
Heart (mg)	253 ± 6	245 ± 5	273 ± 14	268 ± 7	n.s.	P = 0.007	n.s.
Heart:bw	4.5 ± 0.1	4.5 ± 0.1	4.7 ± 0.1	4.8 ± 0.2	n.s.	n.s.	n.s.
***Renal parameters***							
Urine output (ml/24 h)	1.73 ± 0.3	1.35 ± 0.3	2.01 ± 0.4	2.19 ± 0.5	n.s.	n.s.	n.s.
Water consumption (ml/bw/24 h)	0.11 ± 0.008	0.14 ± 0.03	0.26 ± 0.05	0.19 ± 0.01	n.s.	P = 0.0003	n.s.
Albumin excretion (µg/24 h)	9.7 ± 2.1	22.4 ± 3.8	8.2 ± 2.8	27.0 ± 13.0	P = 0.04	n.s.	n.s.
U_Na_V (µmol/24 h)	133 ± 24	99 ± 27	400 ± 70	513 ± 52	n.s.	P < 0.0001	n.s.
U_Cl_V (µmol/24 h)	154 ± 57	130 ± 89	374 ± 143	513 ± 145^*^	n.s.	P < 0.0001	P = 0.04
U_K_V (µmol/24 h)	219 ± 23	210 ± 41	217 ± 37	228 ± 39	n.s.	n.s.	n.s.
Plasma cystatin C (ng/mL)	768 ± 103	880 ± 109	645 ± 46	600 ± 30	n.s.	P = 0.02	n.s.
**FEMALES**							
***Body and organ weights***							
Bw (g)	53 ± 2	50.8 ± 4	54 ± 3	49 ± 3	n.s.	n.s.	n.s.
Kidney (mg)	493 ± 10	481 ± 22	543 ± 37	582 ± 44	n.s.	P = 0.02	n.s.
Kidney:bw	9.7 ± 0.7	9.1 ± 0.4	9.0 ± 0.4	10.4 ± 1.0	n.s.	n.s.	n.s.
Heart (mg)	194 ± 13	189 ± 8	215 ± 13	220 ± 13	n.s.	P = 0.009	n.s.
Heart:bw	3.8 ± 0.4	3.6 ± 0.2	3.6 ± 0.1	3.9 ± 0.3	n.s.	n.s.	n.s.
***Renal parameters***							
Urine output (ml/24 h)	1.50 ± 0.3	1.57 ± 0.3	2.13 ± 0.3	3.02 ± 0.7	n.s.	n.s.	n.s.
Water consumption (ml/bw/24 h)	0.07 ± 0.005	0.074 ± 0.02	0.26 ± 0.05	0.18 ± 0.07	n.s.	P = 0.01	n.s.
Albumin excretion (µg/24 h)	51.5 ± 15.3	57.4 ± 18.0	39.6 ± 16.5	11.8 ± 5.1	n.s.	n.s.	n.s.
U_Na_V (µmol/24 h)	111 ± 21	76 ± 12	534 ± 101	479 ± 98	n.s.	P < 0.0001	n.s.
U_Cl_V (µmol/24 h)	158 ± 27	130 ± 21	546 ± 95	489 ± 92	n.s.	P < 0.0001	n.s.
U_K_V (µmol/24 h)	186 ± 26	170 ± 25	162 ± 24	120 ± 18	n.s.	n.s.	n.s.
Plasma cystatin C (ng/mL)	1246 ± 270	1166 ± 307	785 ± 58	866 ± 78	n.s.	P = 0.07	n.s.

Values are mean ± SEM. Body weight (bw). Body/organ weights: N = 1–2 offspring from 11 litters/group. Renal parameters: male, N = 6–10/group; female, N = 5–10/group. Effect of treatment, diet or interaction (treatment x diet) was evaluated by two-way ANOVA. ^*^Sidak *post hoc* comparing control/hypoxia offspring fed the high salt diet. Not significant (n.s.).

### Offspring body/organ weights at 12 months of age

Body, kidney and heart weights (absolute and corrected for body weight) were similar among control and hypoxia groups at 12 months of age (Table 2). Kidney and heart weight in both sexes were increased by the HS diet compared to the NS diet (Table 2). Kidney to body weight ratio was elevated in male offspring fed HS (Table 2).

### Cardiac histopathology at 12 months of age

**Figure 3:**
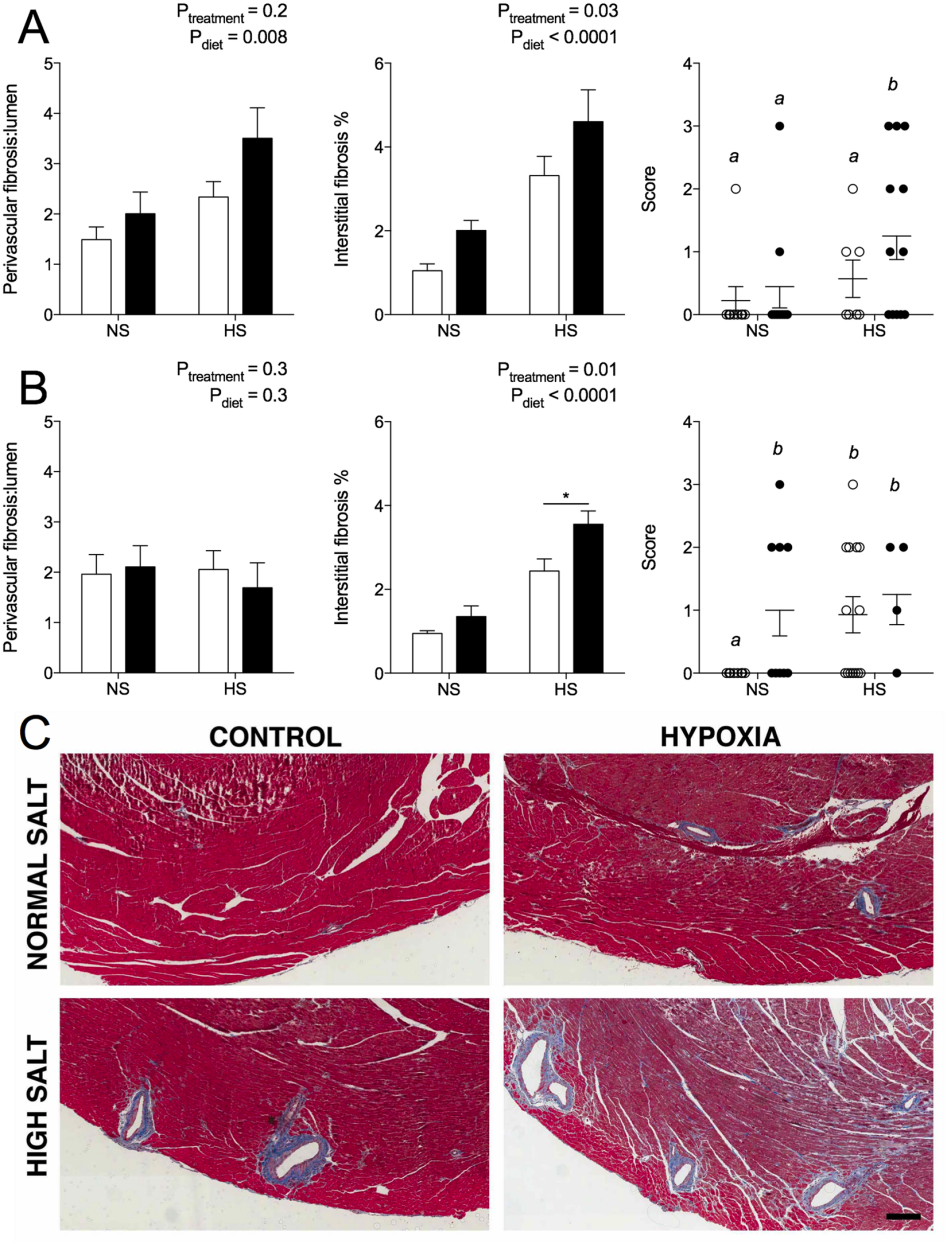
Cardiac histopathology of offspring at 12 months of age. Perivascular fibrosis area normalised to lumen area, interstitial fibrosis expressed as percentage of cardiac tissue, and histology score in male (**A**) and female (**B**) offspring. (**C**) Masson’s Trichrome staining of cardiac tissue in males. Blue staining marks collagen (fibrosis). Scale bar represents 200 µm. Scoring analysed via one-way ANOVA with letters denoting statistical differences between groups. Perivascular and interstitial fibrosis analysed via two-way ANOVA. *P < 0.01 by Sidak *post hoc*. Values are mean ± SEM. Male: N = 5–11/group; female: N = 4–8/group. Control: open bars/points; hypoxia: closed bars/points.

Cardiac perivascular fibrosis was similar between control and hypoxia-exposed male animals, but increased in males fed HS (Fig. 3A,C). Perivascular fibrosis was unaffected by hypoxia and HS in female offspring (Fig. 3B). Interstitial fibrosis was significantly elevated in cardiac tissue by HS in both sexes compared to offspring fed the NS diet, and this effect was greatest in animals exposed to prenatal hypoxia (Fig. 3A–C). Overall cardiac pathology scores of hypoxia-exposed male offspring fed HS were greater than scores of other male groups (Fig. 3A). In females, both prenatal hypoxia and HS increased cardiac pathology scores compared to control offspring fed the NS diet (Fig. 3B). Mild myocardial hypertrophy and interstitial leukocyte infiltration were occasionally observed in the hypoxia-exposed animals fed the NS and HS diets (data not shown).

### Histopathology of kidneys at 12 months of age

**Figure 4:**
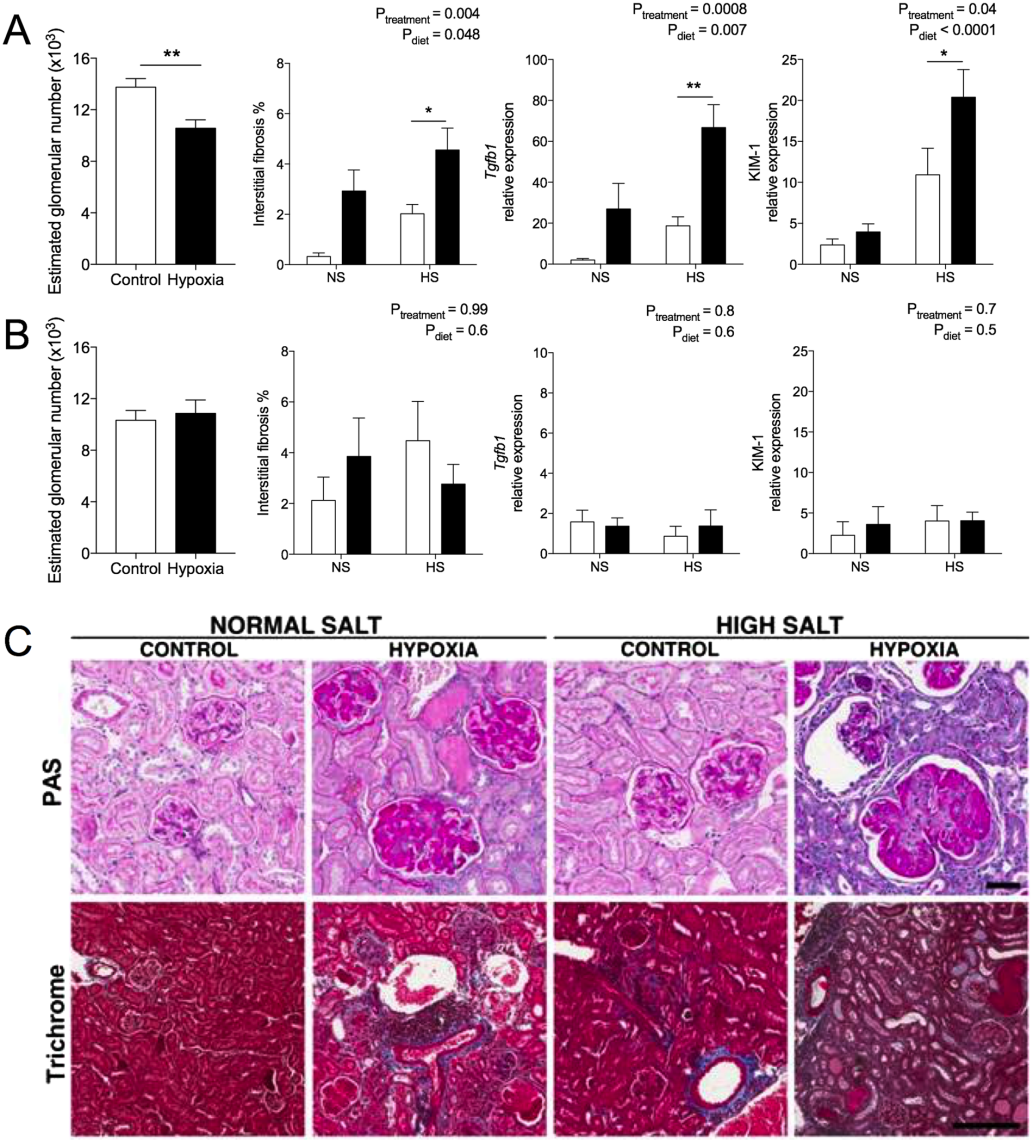
Renal histopathology of offspring at 12 months of age. Estimated glomerular number, interstitial fibrosis expressed as a percentage of renal tissue, and relative mRNA expression of *Tgfb1* and *Kim*-*1* in male (**A**) and female (**B**) offspring. (**C**) Period acid Schiff’s staining of the renal cortex containing glomeruli (scale bar represents 50 µm) and Masson’s Trichrome staining of renal fibrosis (blue) in male offspring (scale bar represents 100 µm). Glomerular number was analysed using a Student’s t test (**P = 0.007). Interstitial fibrosis and mRNA expression analysed via two-way ANOVA. Values are mean ± SEM. Male: N = 5–11/group; female: N = 6–8/group. Control: open bars; hypoxia: closed bars.

Similar to findings at P21, there was a nephron deficit of 25% in male (Fig. 4A) but not female-hypoxia offspring at 12 months of age (Fig. 4B). Cross-sectional glomerular tuft area was ~25% greater in male hypoxia-exposed offspring compared to male controls (control NS: 4069 ± 206, hypoxia NS: 5100 ± 471 μm^2^; P_treatment_ = 0.03). The HS diet increased glomerular area in all animals, compared to offspring fed NS (control HS: 5009 ± 331, hypoxia HS: 5702 ± 329 μm^2^; P_diet_ = 0.048). Perivascular fibrosis area tended to be greater in male hypoxia-exposed offspring, with no effect of HS, although this did not reach statistical significance (control NS: 2.2 ± 0.6, hypoxia NS: 3.6 ± 0.9, control HS: 2.4 ± 0.2, hypoxia HS: 3.2 ± 0.4; P_treatment_ = 0.08, P_diet_ = 0.8). Renal interstitial fibrosis was greater in male hypoxia-exposed offspring compared to controls, and further increased by the HS diet (Fig. 4A).

Hypoxia-exposed male offspring had increased renal *Kim*-*1* mRNA expression compared to control offspring (Fig. 4A), and this was further increased by the HS diet. Male kidneys from hypoxia-exposed offspring fed the NS and HS diets had greater overall pathology scores than control counterparts (see Supplementary Fig. S4A); overall pathology scores for female kidneys did not differ between treatment or dietary groups (see Supplementary Fig. S4B). Histologically, the kidneys from hypoxia-exposed male offspring were characterized by widespread, mild to severe interstitial fibrosis, increases of mesangial matrix and glomerular basement membranes, mild to moderate thickening of the basement membrane of the Bowman’s capsule, segmental thickening of tubular basement membranes, occasional regeneration of tubules and presence of hyaline, and acellular casts in tubular lumina (Fig. 4C). These changes were exacerbated in animals fed HS. In most kidneys of hypoxia-exposed male offspring there was multifocal, mild to moderate interstitial infiltration of lymphocytes and fewer macrophages; often with a clear vasculocentric localisation (Fig. 4C).

In females, hypoxia and HS had no effect on glomerular area (control NS: 4893 ± 559, hypoxia NS: 3928 ± 344, control HS: 5213 ± 750, hypoxia HS: 4295 ± 398 μm^2^), perivascular fibrosis (control NS: 1.9 ± 0.6, hypoxia NS: 1.9 ± 0.6, hypoxia NS: 2.3 ± 0.5, hypoxia HS: 1.7 ± 0.40), interstitial fibrosis or mRNA expression of *Kim1* and *Tgfb1* (Fig. 4B). When male and female offspring were examined together, female offspring had significantly increased albumin excretion and a tendency towards reduced increased plasma cystatin C (see Supplementary Fig. 6). This suggests glomerular filtration rate was lower in females compared to males, irrespective of prenatal treatment.

### Correlation analysis of blood pressure and renal outcomes at 12 months of age

We then examined whether elevations in blood pressure were associated with the adverse renal outcomes reported in 12-month-old offspring fed the normal salt diet (Table 3). Renal renin content was correlated with DBP and MAP in males. In females, renal renin content was correlated with SBP and MAP; renal renin trended towards an association with DBP. We identified that MAP and DBP were both correlated with albumin excretion, glomerular hypertrophy and renal interstitial fibrosis in male offspring, but were not correlated with cardiac interstitial fibrosis. Female offspring had an association between albumin excretion and MAP; no associations were observed between blood pressures and glomerular hypertrophy and renal interstitial fibrosis. Cardiac interstitial fibrosis was correlated with SBP, DBP and MAP in female offspring only. No association between plasma cystatin C and blood pressures were observed in either sex (data not shown).

**Table 3:** Correlation analysis of blood pressure and renal/cardiac parameters in 12-month-old offspring.

	Renal renin content	Albumin	Glomerular area	Renal interstitial fibrosis	Cardiac interstitial fibrosis
***Males***					
*Systolic blood pressure*	No correlation	No correlation	No correlation	No correlation	No correlation
*Diastolic blood pressure*	r^2^ = 0.60; P = 0.008	r^2^ = 0.63; P = 0.004	r^2^ = 0.73; P = 0.002	r^2^ = 0.80; P = 0.0005	No correlation
*Mean arterial pressure*	r^2^ = 0.40; P = 0.04	r^2^ = 0.60; P = 0.003	r^2^ = 0.65; P = 0.003	r^2^ = 0.59; P = 0.006	No correlation
***Females***					
*Systolic blood pressure*	r^2^ = 0.59; P = 0.004	No correlation	No correlation	No correlation	r^2^ = 0.86; P = 0.01
*Diastolic blood pressure*	r^2^ = 0.32; P = 0.07	No correlation	No correlation	No correlation	r^2^ = 0.66; P = 0.01
*Mean arterial pressure*	r^2^ = 0.59; P = 0.01	r^2^ = 0.42; P = 0.03	No correlation	No correlation	r^2^ = 0.57; P = 0.03

### Histopathology of kidneys at 4 months of age

We then examined kidneys of 4-month-old males to determine whether pathological changes due to prenatal hypoxia or HS were evident at a younger age. Urine output and electrolyte excretion were increased by the HS diet, but were not affected by hypoxia (see Supplementary Fig. S1). The HS diet increased perivascular fibrosis in all offspring compared to offspring fed NS (see Supplementary Fig. S5A,B). Interstitial fibrosis was increased in hypoxia-exposed male offspring, as well as offspring fed the HS diet (see Supplementary Fig. S5A,B).

## Discussion

Our study demonstrates that prenatal hypoxia exposure is associated with elevated blood pressure, cardiac fibrosis and temporal activation of the renal RAS in both sexes; however, significant renal impairments were only observed in male offspring. Hypoxia reduced the number of glomeruli in male offspring, and this was associated with glomerular hypertrophy, renal fibrosis and increased albumin excretion by 12 months of age. A postnatal high salt diet caused a rapid and severe exacerbation of renal fibrosis in male offspring exposed to prenatal hypoxia. In contrast, female offspring exposed to the same hypoxic insult had no signs of renal impairment compared to control females. Postnatal high salt intake exacerbated cardiac fibrosis in both sexes exposed to prenatal hypoxia. The renal RAS was activated in males exposed to prenatal hypoxia from early in life with changes evident in females only occurring with aging. Together, our study shows prenatal hypoxia predisposes both sexes to cardiovascular impairments, with renal deficits limited to male offspring. This suggests the female sex affords some form of reno-protection during the hypoxic insult, which is not extended to the cardiovascular system.

In humans, fetal hypoxia results in asymmetric growth restriction and low birth weight^4, 24^, both of which are associated with hypertension and cardiovascular diseases in adulthood^1^. In our model, male and female hypoxia-exposed offspring are growth restricted with significantly smaller kidneys both at late gestation and weaning. Prenatal hypoxia results in redistribution of cardiac output away from peripheral tissues towards the heart and brain^25^, which may explain why kidneys of hypoxia-exposed animals are lighter than controls but heart and brain weights are maintained. Furthermore, reduced blood supply may contribute to the 25% reduction in glomeruli in kidneys of male hypoxia-exposed offspring. This glomerular deficit is equivalent to that seen in mice exposed to transient mid-gestational hypoxia (although in that study, data from males and females were combined)^26^ and male offspring following uteroplacental insufficiency in the rat^16^. However, in the present study, female hypoxia-exposed offspring had a similar glomerular number to female controls. Although surprising, our finding is not unprecedented, as both modest protein restriction in the rat^27, 28^ and birth asphyxia in the spiny mouse^29^ reduce glomerular number in male offspring only. Together, these studies highlight that the developing kidney of male fetuses is more vulnerable to prenatal insults than the kidney of females.

A nephron deficit is associated with hypertension in the human population^15^ and animal models^2^. Consequently, we hypothesised that only male offspring would develop high blood pressure. However, both sexes had elevated blood pressure at 12 months of age, highlighting dissociation between nephron number and hypertension. Endothelial dysfunction and impaired vascular structure (as previously reported in both sexes exposed to hypoxia)^8^ could contribute this increase in blood pressure. Furthermore, diastolic blood pressure was elevated in both male and female mice, consistent with increased peripheral vascular resistance of which microvascular endothelial dysfunction^8^ could be a contributing factor. In contrast to males, female hypoxia-exposed offspring also had elevated systolic pressure. Clinically, isolated diastolic hypertension is more predominant in young males compared to the population as a whole, and appears to be antecedent to systolic hypertension^30^.

Alterations in the renin-angiotensin system (RAS) are a major contributor to hypertension, and changes to the renal RAS occur following a range of *in utero* perturbations^20^. Therefore, we hypothesised that elevated blood pressure in male and female hypoxia-exposed offspring would be associated with alterations in the renal RAS. Indeed, increased renal *renin* mRNA expression was present in male, but not female, hypoxia-exposed mice at just three weeks of age. By 12 months, renal *renin* mRNA and renin content were increased in both sexes exposed to prenatal hypoxia, with renin content directly associated with elevated diastolic blood pressures in both sexes. This may directly contribute to elevated blood pressure given persistent upregulation of renin production is associated with hypertension in growth-restricted offspring exposed to uteroplacental insufficiency^21^ and protein restriction^31^. Male (but not female) hypoxia-exposed offspring also had increased expression of renal *At1aR* mRNA. This combined with increased renal renin content suggests that there is early and sustained activation of the intrarenal RAS in males following hypoxia. Other models of prenatal hypoxia (including those resulting from placental insufficiency) have demonstrated increased renal sympathetic nerve activity^32^ and increased vascular tone (in part through enhanced endothelin signalling)^33^ contribute to high blood pressure in young male rodents only. These changes are likely to occur secondary to changes in the RAS as proposed recently by Dasinger and colleagues^19^. Considering the increase in intrarenal renin content in aged males and females exposed to hypoxia, future studies should examine if RAS blockade is an effective anti-hypertensive therapy for both sexes in this model as previously shown in rat offspring following placental insufficency^21^.

Temporal differences in intrarenal RAS activation between the sexes suggests that male hypoxia-exposed offspring may have developed elevated blood pressure at an earlier age to females in our study. As hypothesized recently^19^, the onset of hypertension in female rodents following a prenatal perturbation occurs in middle/old age (>12 months old). Estradiol is protective against cardiovascular risk in young female offspring following hypoxia due to placental insufficiency, however blood pressure elevation occurs around middle age following early reproductive senescence and RAS activation^34, 35^. Similarly in the human population, hypertension is highly prevalent in postmenopausal women^36^. Future studies in our model should examine the time point of reproductive senescence and blood pressure elevation in offspring exposed to prenatal hypoxia.

A novel aspect of our study was the in-depth morphological analysis, in particular the development of renal and cardiac fibrosis, following prenatal hypoxia. Significant interstitial fibrosis was observed in the hearts of both sexes exposed to prenatal hypoxia, which is associated with reduced myocardial and arterial compliance^37^. Pathological myocardial fibrosis is associated with diastolic dysfunction, and although we did not perform echocardiography in our study, a similar model of prenatal hypoxia (12% O_2_, E15–birth) showed that rat offspring had normal cardiac function at 4 months but developed left ventricular diastolic dysfunction at 12 months^38^. This is consistent with our morphological findings in the mouse, and suggests our hypoxia-exposed animals may be at risk of diastolic dysfunction and heart failure. Although we were unable to measure blood pressure in the animals fed the high salt diet, cardiac interstitial fibrosis was exacerbated in animals on this diet. Indeed, in females, the correlation between blood pressure and cardiac interstitial fibrosis suggests the high salt diet may exacerbate the elevations in blood pressure observed in hypoxia-exposed offspring. Furthermore, increased perivascular fibrosis, which is associated with impaired coronary blood flow in humans^39^, was most evident in hypoxia-exposed males fed the high salt diet. Together these findings suggest that the additional postnatal high salt diet may further increase risk of cardiovascular diseases in both sexes.

The most profound finding of this study was the overt renal pathology observed in hypoxia-exposed male offspring fed normal salt, including progressive glomerular hypertrophy, glomerulosclerosis, tubulointerstitial fibrosis and perivascular fibrosis from early life until 12 months of age. Furthermore, mild albuminuria and elevated renal mRNA expression of the tubular injury marker *Kim*-*1* was evident at 12 months, which is indicative of progressive renal damage given no pathological changes were observed at 4 months of age. Our correlation analyses revealed elevated mean arterial pressure in male offspring was correlated with glomerular hypertrophy, suggesting offspring fed high salt were at increased risk of hypertension and subsequent renal injury. Albuminuria in humans is associated with cardiovascular risk factors such as vascular endothelial dysfunction^40^ which is present in our model at 12 months of age^8^. Fibrosis is also a hallmark of aging, and is present in pathological situations such as cardiovascular and kidney disease^41^. The AT1 receptors are known mediators of fibrosis, in part through the TGF-β1 pathway. Renin also initiates the production of pro-fibrotic factors (primarily TGF-β1) through the prorenin receptor, independent of angiotensin II^42^. Given renin content and *At*_*1a*_ *R* and *Tgfb1* mRNA expression were enhanced in male hypoxia-exposed offspring, this may be an underlying molecular mechanism for the widespread renal fibrosis in this group. Intriguingly, all female offspring had greater intrarenal renin content, greater albumin excretion and a tendency towards increased plasma cystatin C (indicative of reduced GFR) compared to control males at 12 months of age. No signs of renal impairments were observed in younger female offspring, suggesting these deficits emerged with age. This could be associated with naturally lower nephron endowment in female offspring compared to males. There was no effect of prenatal treatment, which may be due in part to the fact that glomerular number was not altered in female offspring exposed to hypoxia, allowing maintenance of renal function equivalent to control counterparts across the lifespan.

The combination of prenatal hypoxia and the challenge of a chronic high salt diet in postnatal life was strongly associated with marked elevations in mRNA expression of *Kim*-*1* and *Tgfb1* in combination with increased glomerulosclerosis and fibrosis in the male offspring at 12 month of age. Consequently, we examined kidneys from males at 4 months of age to determine whether fibrosis was present at an earlier age. Strikingly, significant interstitial renal fibrosis was evident in young hypoxia-exposed male offspring within six weeks of high salt diet consumption, suggesting the combination of prenatal and postnatal insults markedly increases the rate of progression of renal damage. Given perivascular fibrosis was mainly influenced by diet but not prenatal treatment, consumption of a normal salt diet throughout life substantially attenuated renal damage in male hypoxia-exposed offspring. Glomerulosclerosis and renal fibrosis, in conjunction with hypertension, are hallmarks of chronic kidney disease. Therefore, we measured plasma cystatin C, currently the best endogenous marker of GFR in humans^43^, to achieve an indication of renal function in this study. In animals fed high salt, a reduction in plasma cystatin C was observed. This indicates glomerular hyperfiltration, a common response to nephron loss and suggested to promote glomerular hypertrophy and subsequent sclerosis^44^. Whether a sustained low salt diet from early in life could prevent the development of renal fibrosis is a pertinent clinical question given children over 5 years of age are commonly consuming >100 mmol/day of sodium, 5–10 times the physiological requirement^45^.

We have shown that organ systems controlling blood pressure and fluid homeostasis, namely the kidney, heart and vasculature, are impaired by prenatal hypoxia. We conclude that elevated blood pressure and associated cardiac/renal injury observed in male offspring exposed to hypoxia is likely associated with the reduction in nephron number and activation of the renal RAS in early life. These sequelae were further exacerbated by a high salt diet. In females, the same prenatal hypoxic insult led to elevated blood pressure, an increase in intrarenal renin, and susceptibility to salt-induced cardiac injury in the absence of a nephron deficit. This suggests that the kidneys of female offspring were protected from the *in utero* hypoxic insult; however, this protection does not extend to the cardiovascular system. Further investigation into the mechanisms underlying the sex differences in renal outcomes following prenatal hypoxia are needed and may contribute to a better understanding of renal disease progression and treatment. Temporal differences in renal RAS activation may be a contributing factor, and therefore future experiments could investigate whether RAS blockade in early life may be an effective anti-hypertensive therapy and ameliorate end-organ damage observed in these offspring. Given our findings, we suggest curtailing salt intake can minimise progressive cardiac and renal damage for those known to have suffered from an adverse *in utero* environment.

## Methods

### Maternal hypoxia

All experiments were approved by the University of Queensland Animal Ethics Committee and were conducted in accordance with the *Australian Code of Practice for the Care and Use of Animals for Scientific Purposes*. Time-mated CD1 mice at embryonic day (E) 14.5 of pregnancy, were randomly allocated to normoxic room conditions (N = 19) or housed inside a hypoxic chamber continuously flushed with nitrogen gas to maintain an oxygen concentration of 12% (N = 19)^8^. A 12 h light/dark cycle was maintained and food and water was provided *ad libitum*. A subset of dams (N = 8) was culled at E18.5^6^. Fetal body weight, kidney, heart and brain weights were recorded. Remaining dams were removed from the hypoxic chamber upon littering down. Pup weights were monitored daily from P1 until weaning at P21. At P21, a subset of male and female offspring (1–2 of each sex per litter) was euthanized by cervical dislocation. Kidneys, hearts and brains were dissected and weighed. The left kidney was fixed in 4% paraformaldehyde (PFA) for assessment of renal pathology, glomerular number and area. The remaining offspring were aged to 12 months for renal and cardiovascular studies.

### High salt diet

A subset of animals aged 10 weeks (N = 11 per sex per treatment, one or two animals from each litter) was randomly allocated to receive a high salt (HS) diet (5% NaCl, wt/wt; modified AIN 93 M, SF05-023; Specialty Feeds, Glen Forrest, WA, Australia) whilst litter mates remained on a normal salt (NS) diet (0.26% NaCl, wt/wt; AIN93M; Specialty Feeds) as previously described^8^. Animals were maintained on the diet until euthanasia at 12 months of age.

### Estimation of glomerular number at P21

A combined stereological-histochemical approach was used to determine glomerular number (N = 8 per treatment group) as previously described^46^. Briefly, kidneys were processed into paraffin blocks and exhaustively sectioned at 5 µm. Ten evenly spaced section pairs were systematically sampled and stained with lectin peanut agglutinin (Arachis hypogea, PNA; Sigma Aldrich, Castle Hill, NSW, Australia). Glomeruli from each section pair were counted using the physical disector/fractionator combination.

### Blood pressure

Animals at 12 months of age, fed the normal salt diet throughout life, were anaesthetised under isoflurane (3–3.5% in oxygen, ~125 ml min^−1^) and radiotelemetry transmitters (model PA-C10; Data Sciences International, MN, USA) were implanted as described previously^47^. Recording of heart rate (HR), systolic (SBP) and diastolic blood pressure (DBP), and activity data commenced in conscious, unrestrained animals 10 days post-surgery (N = 5–6 per treatment group). Data were acquired for 10 seconds every 15 minutes for three days. Mean arterial pressure (MAP) and pulse pressure (PP) were calculated from these parameters.

### Restraint stress

On the fourth day of measurements, animals implanted with radiotelemeters were subjected to a restraint stress challenge. Data was acquired for 10 s every 5 min for 1 h before restraint stress, and averaged to establish a baseline. Animals were immediately placed in a clear plastic container (12 × 8 × 6 cm) for 15 min, and then released to the home cage. Data was acquired continuously during the restraint and 15 min recovery period. Recovery data was sampled for 10 s every 5 min for the next hour of recovery.

### Urinalysis

Male and female offspring at 2 months (normal salt animals only, N = 6 per treatment group), 4 months of age (N = 6–8 per treatment group) and 12 months (N = 9–11 per treatment group) fed the normal salt and high salt diets, respectively, were acclimatised to individual metabolic cages prior to urine collection. Animals were then placed in individual metabolic cages for 24 h. Urine was collected and frozen at −20 °C. Urinary electrolytes (Na^+^, K^+^, Cl^−^) were measured using a COBAS Integra 400 Plus analyser. Urinary excretion of albumin was determined in duplicate using commercially available mouse kits (Albuwell M, Exocell, Philadelphia, USA). In addition, water consumption in home cages was measured over 7 days at 12 months of age and averaged per day.

### Post-mortem tissue collection and histopathology

Following all measurements, animals were euthanized via carbon dioxide inhalation. Blood was collected via cardiac puncture and plasma separated by centrifugation. Body and organ weights were recorded. Hearts and kidneys were processed to paraffin as described above. Representative 5 µm midline sections from each heart and kidney were stained with Periodic Acid Schiff’s (PAS) and Masson’s Trichrome. Sections were assessed by an expert pathologist blinded to treatment groups and assigned a score based on pathology severity: 0, normal; 1, minimal change; 2, mild; 3, moderate; 4, severe. Glomerular area in PAS-stained kidney sections was quantified by tracing glomerular borders when the vascular pole was evident. Twenty to 30 glomeruli were analysed per animal and measurements averaged. To assess perivascular fibrosis in kidneys and hearts, two arterioles were selected in the Masson’s trichrome-stained sections, as described in ref. 48. Area of adventitial collagen was measured and normalised to vessel lumen area, and averaged for each animal. Interstitial fibrosis was quantified by determining percentage of collagen in the interstitium in four random fields of view per animal.

### Plasma analysis

Plasma cystatin C levels at 12 months of age (1:300 dilution) were determined in duplicate using a commercially available kit (Mouse/Rat Cystatin C Quantikine ELISA kit, R&D Systems).

### mRNA expression

RNA was extracted from whole kidneys from offspring at P21 (N = 9–11 per group) and 12-month-old offspring (N = 7–11 per group) using the RNeasy minikit (QIAGEN, Chadstone Centre, VIC, Australia). All RNA was treated with deoxyribonuclease 1 and reverse transcribed into cDNA (iScript^TM^, Bio-Rad, Gladesville, NSW, Australia). Taqman assays on demand (Life Technologies, Mulgrave, VIC, Australia) were used to determine mRNA levels of *Renin* (Mm0234887_mh), *Ace* (angiotensin converting enzyme, Mm00802048_m1) and *Kim*-*1* (kidney injury marker 1, Mm00506686_m1). Custom probes and primers to detect *AT*_*1a*_ *R* (angioteninsin type 1a receptor) and *Tgfb1* (transforming growth factor beta 1) mRNA levels were used as previously described^7, 49^. The comparative cycle threshold method was used for all expression assays using the *B*-*actin* endogenous control. mRNA levels were normalised to the mean of the control male group at P21, or the mean of the control group of their own sex at 12 months of age.

### Renal tissue RAS content

Renal tissue from homogenised in radioimmunoassay buffer (N = 7–11/group) and tissue renin, angiotensin II (Ang II) and angiotensin 1–7 (Ang 1–7) concentrations were measured by radioimmunoassay as previously described^50^.

### Statistical analysis

Statistical analyses were performed using GraphPad Prism 7 and IBM SPSS. Data are presented as mean ± standard error of the mean (SEM). Multivariate analysis of variance (MANOVA) was used to analyse blood pressure recordings over three days. Prenatal treatment, sex and light-dark periods were assigned as independent variables. Restraint stress response was calculated by quantifying the area under the curve during the stressor and comparing with an equivalent amount of time during the baseline period. Histology scoring was analysed using a Mann-Whitney test. Glomerular number was analysed using a two-tailed, unpaired Student’s *t*-*test*. A Pearson’s correlation coefficient was also used to determine the relationship between blood pressure radiotelemetry recordings and renal/cardiac parameters in offspring fed the normal salt diet at 12 months of age. All remaining data were analysed using two-way ANOVA with a Sidak *post hoc* test used where appropriate. P < 0.05 was considered significant.

### Data availability

The datasets generated during and/or analysed during the current study are available from the corresponding author on reasonable request.

## Electronic supplementary material

Supplementary Data 1
