# Supplementary material for: Prenatal hypoxia leads to hypertension, renal renin-angiotensin system activation and exacerbates salt-induced pathology in a sex-specific manner
Source: Sci Rep. 2017 Aug 15;7:8241. doi: 10.1038/s41598-017-08365-4 (PMC5557956; doi:10.1038/s41598-017-08365-4)
Supplement: Supplementary file 1 — Supplementary Data 1 [file 41598_2017_8365_MOESM1_ESM.pdf]

TITLE PAGE

PRENATAL HYPOXIA LEADS TO HYPERTENSION, RENAL RENIN-ANGIOTENSIN SYSTEM ACTIVATION AND EXACERBATES SALT-INDUCED PATHOLOGY IN A SEX-SPECIFIC MANNER

Sarah L Walton[1], Helle Bielefeldt-Ohmann[2], Reetu R Singh[3], Joan Li[4], Tamara M Paravicini[1,5], Melissa H Little[4,6], Karen M Moritz[1,7]

[1]School of Biomedical Sciences, The University of Queensland, St Lucia, Queensland, Australia
[2]School of Veterinary Science, The University of Queensland, Gatton, Queensland, Australia
[3]Department of Physiology, Monash University, Clayton, Victoria, Australia
[4]Institute for Molecular Bioscience, The University of Queensland, St Lucia, Queensland, Australia
[5]School of Health and Biomedical Sciences, RMIT University, Melbourne, Victoria, Australia
[6]Murdoch Childrens Research Institute, Parkville, Victoria, Australia
[7]Child Health Research Centre, The University of Queensland, Australia

SUPPLEMENTAL MATERIALS

Supplementary Table 1

Supplementary Figure S1
Supplementary Figure S2
Supplementary Figure S3
Supplementary Figure S4
Supplementary Figure S5
Supplementary Figure S6

Supplementary Table S1. *P* values of MANOVA of radiotelemetry parameters from 12-month-old offspring.

	SBP	DBP	MAP	PP	HR	Activity
**Treatment**	0.001*	0.001*	0.001*	0.23	0.11	0.13
**Period**	0.002*	0.012*	0.001*	0.28	0.001*	0.001*
**Sex**	0.046*	0.14	0.001*	0.22	0.001*	0.65
**Treatment x sex**	0.013*	0.77	0.039*	0.008*	0.69	0.63
**Treatment x period**	0.98	0.88	0.85	0.71	0.92	0.042*
**Treatment x sex x period**	0.67	0.79	0.86	0.96	0.59	0.90

Online supplemental figures

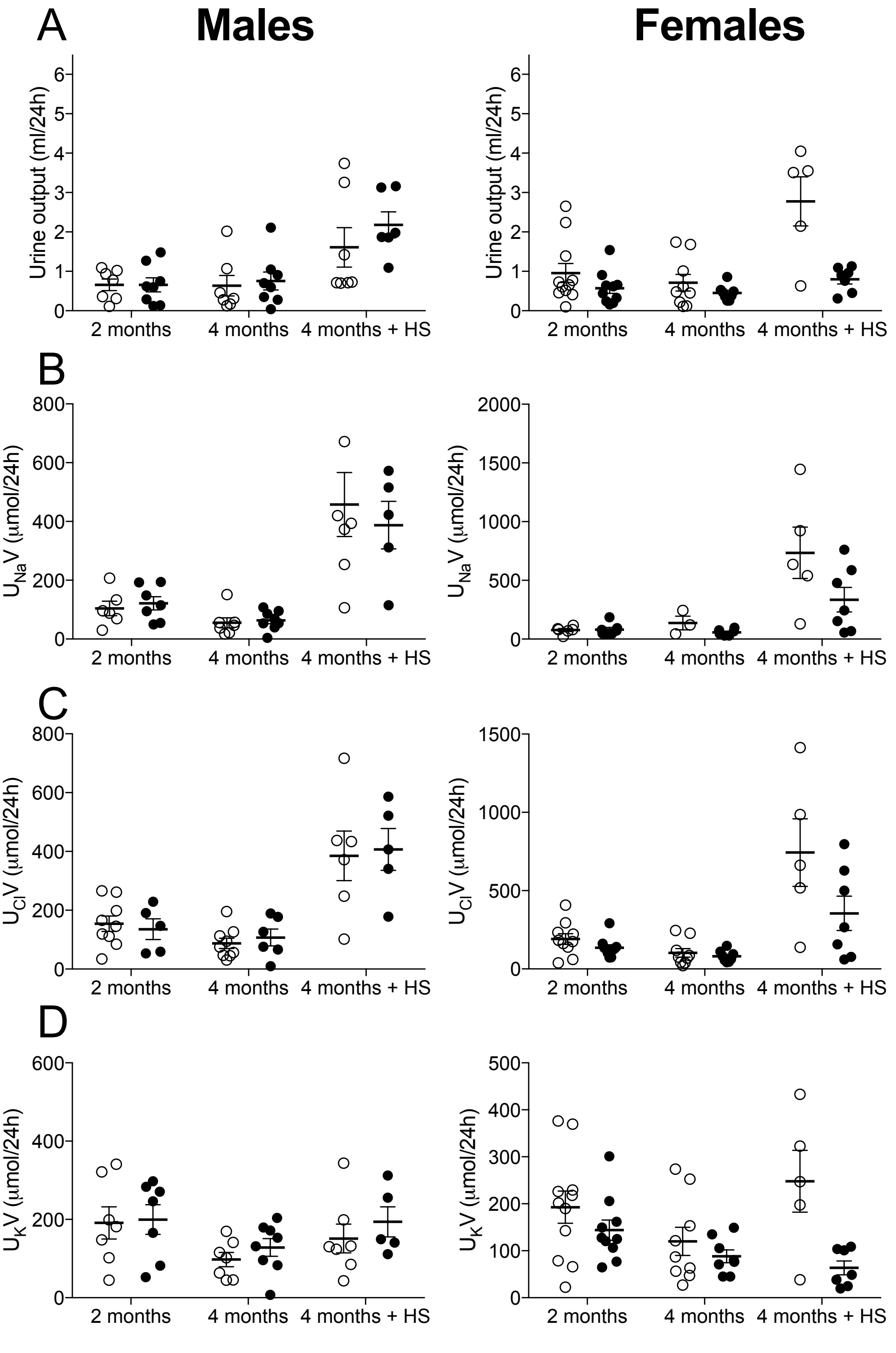

Supplementary Figure S1. Effect of prenatal hypoxia on renal function in offspring aged 2 months, and 4 months (normal salt and high salt [HS] diet).
(A) Urine output (ml/24h), (B) Urinary excretion of sodium (µmol/24h), (C) urinary excretion of chloride (µmol/24h), and (D) urinary excretion of potassium (µmol/24h). Data comparing control and hypoxia-exposed offspring at 2 months of age were analysed by Student's t test. Data from 4-month-old offspring was analysed via two-way ANOVA with prenatal treatment and diet as factors. Control: open circles; hypoxia: black circles. Data expressed as mean ± SEM. Male: N=6-10 animals per group; female: N=5-10 animals per group.

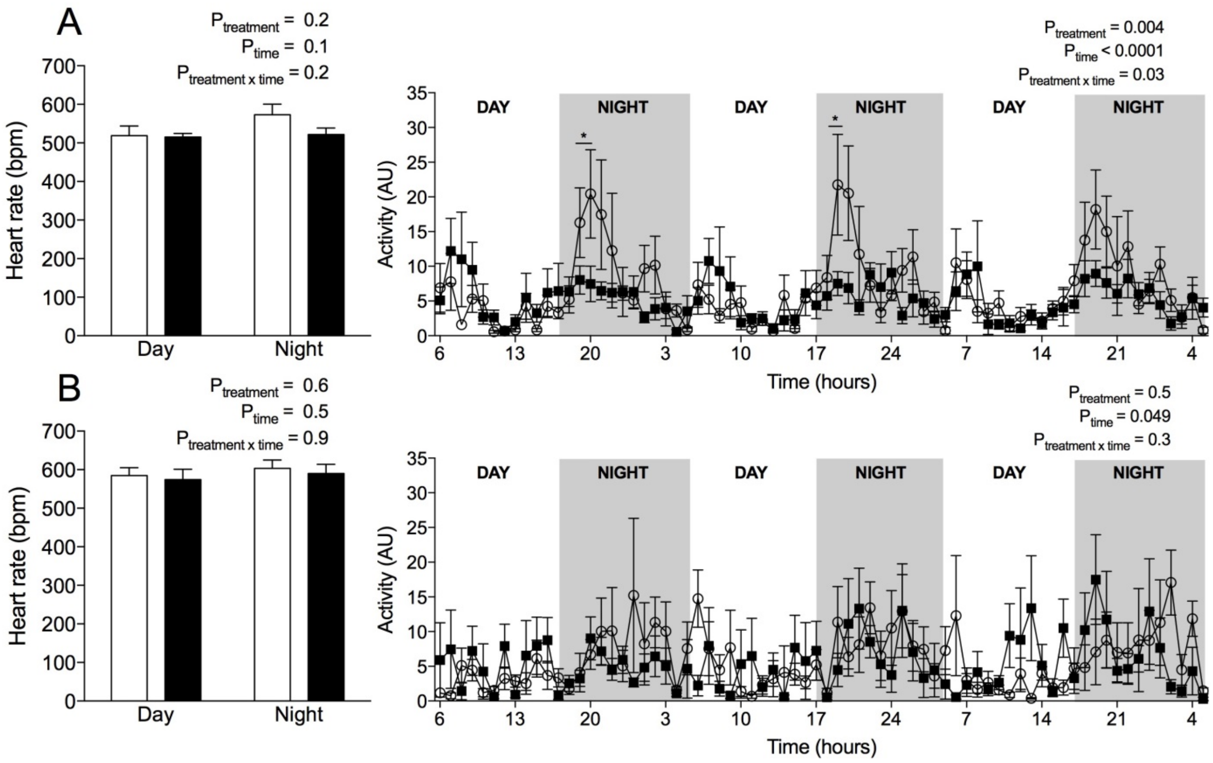

Supplementary Figure S2. Effect of maternal hypoxia on heart rate and activity of offspring at 12 months of age.

Heart rate (bpm, beats per minute) and activity recordings in male (A) and female (B) offspring at 12 months of age. Data was analysed via two-way ANOVA and expressed as mean ± SEM. *P<0.01 by Sidak *post hoc*. Control: white bars/open points; hypoxia: black bars/black points. N=5-6 animals per group.

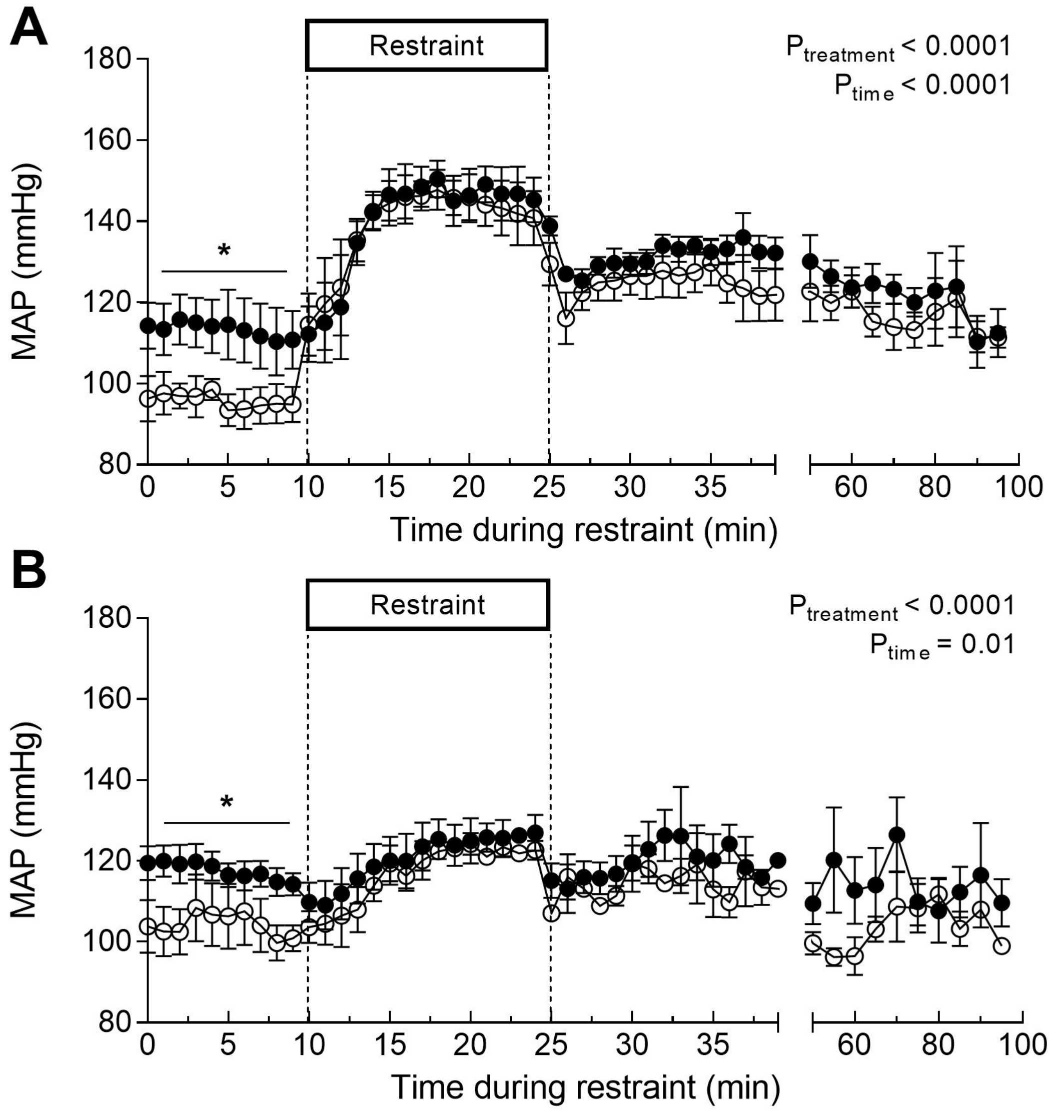

C

	Control		Hypoxia		Two-way ANOVA		
	Male	*Female*	*Male*	*Female*	$P_{treatment}$	P_{sex}	$P_{treatment\ x\ sex}$
SBP (ΔAUC)	1270 ± 175	692 ± 429	879 ± 216	560 ± 139	0.3	0.06	0.6
DBP (ΔAUC)	1004 ± 133	510 ± 356	695 ± 223	437 ± 131	0.4	0.07	0.6
MAP (ΔAUC)	1147 ± 156	561 ± 362	783 ± 209	499 ± 133	0.3	0.049	0.5
PP (ΔAUC)	313 ± 57	216 ± 84	199 ± 33	259 ± 65	0.6	0.8	0.2
HR (ΔAUC)	6438 ± 1076	2315 ± 511	5308 ± 1629	2967 ± 547	0.8	0.02	0.5

Supplementary Figure S3. Mean arterial pressure (MAP, mmHg) response to a 15-minute restraint stress in male (A) and female (B) offspring. (C) Stressor measurements (systolic blood pressure [SBP, mmHg], diastolic blood pressure [DBP, mmHg], MAP [mmHg] and pulse pressure [PP, mmHg]) were calculated by quantifying area under the curve (AUC) during the stressor and normalised to an equivalent amount of time during baseline (pre-stressor). Data were analysed via two-way ANOVA and expressed as mean ± SEM. * P<0.05 by Sidak *post hoc*. Control: white points; hypoxia: black points. Male: N=5-6 animals per group; female: N=3-5 animals per group.

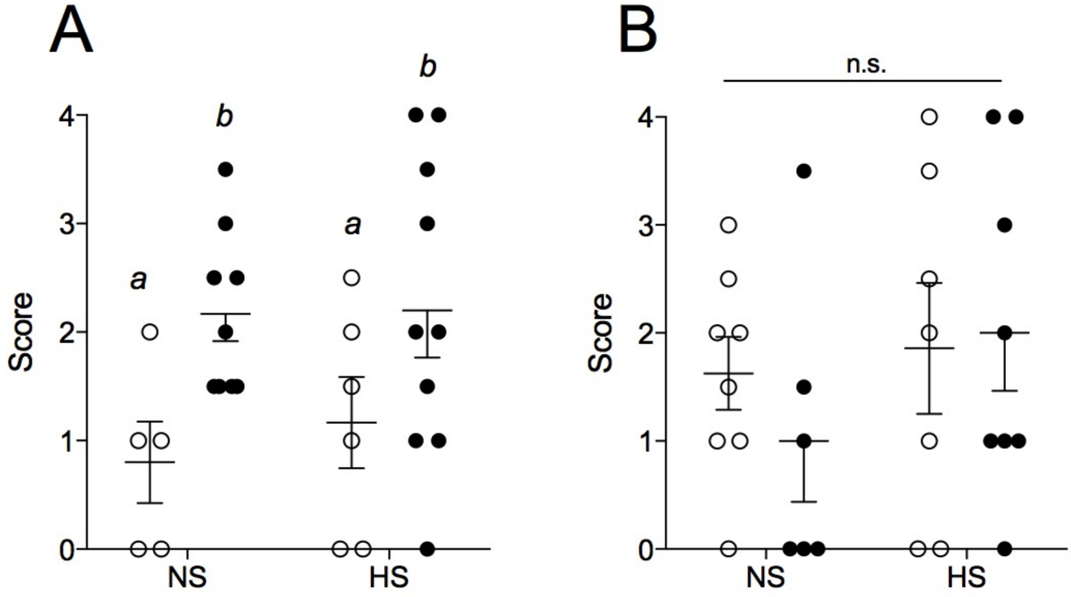

Supplementary Figure S4. Renal histopathology score in offspring at 12 months of age. Overall histology score assigned by pathologist to kidneys from male (A) and female (B) offspring at 12 months of age. Scoring analysed via one-way ANOVA with different letters denoting statistical differences between groups; 'n.s' denotes 'not significant'. Values are mean±SEM. Male: N=5-11/group; female: N=6-8/group. Control: open points; hypoxia: closed points.

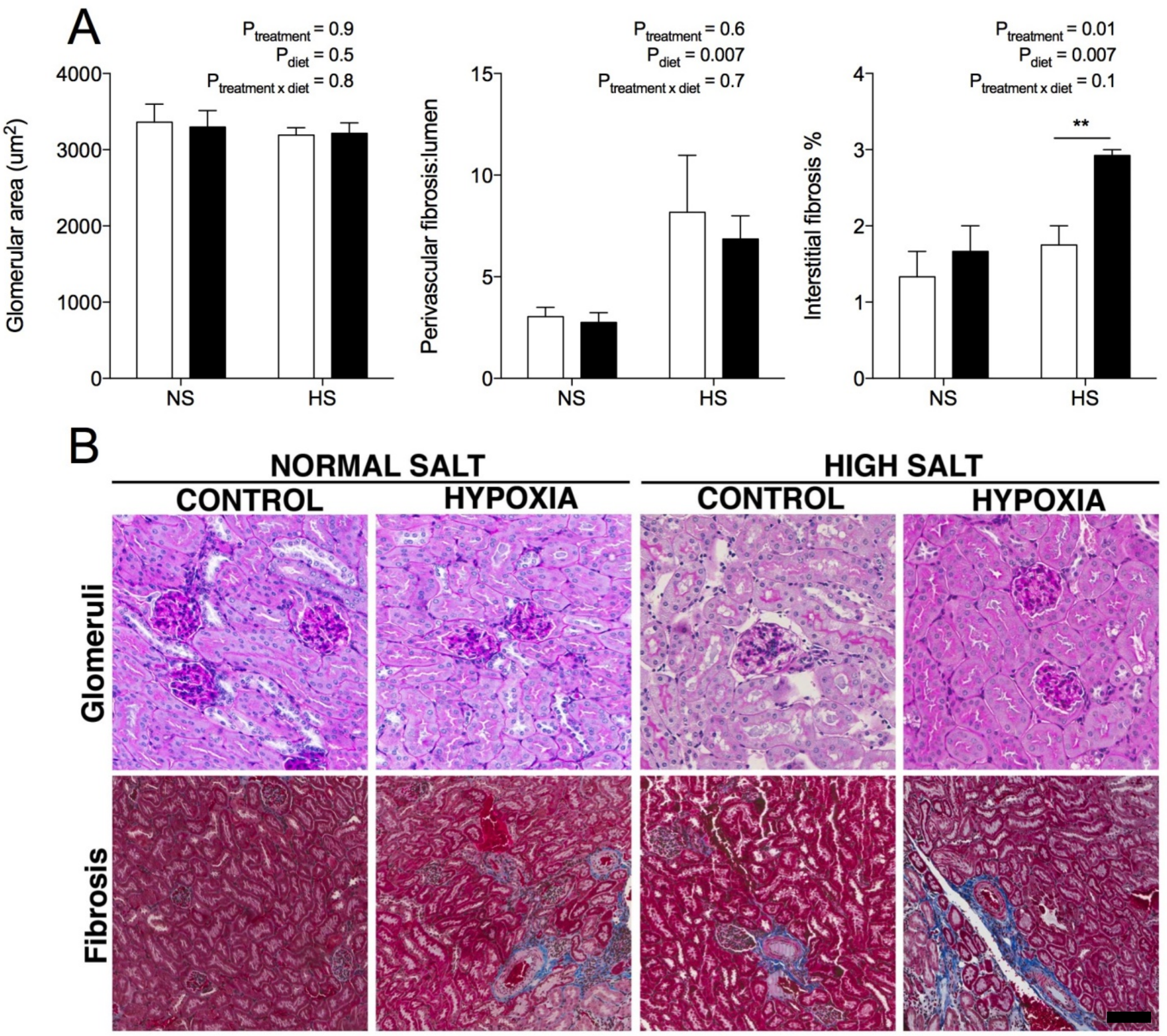

Supplementary Figure S5. Renal morphometry and histopathology in male offspring at 4 months of age.

(A) Glomerular area, perivascular fibrosis area normalised to lumen area, interstitial fibrosis expressed as a percentage of renal tissue, and overall histopathology score. (B) Periodic acid Schiff's staining of the renal cortex containing glomeruli. Masson's Trichrome staining of renal fibrosis (blue) in male offspring. Scale bar represents 200 µm. Scoring was analysed via one-way ANOVA with different letters denoting statistical differences between groups. Perivascular and interstitial fibrosis, and glomerular area were analysed via two-way ANOVA. All data expressed as mean ± SEM. Control: open bars; hypoxia: black bars. Male: N=4-5 animals per group.

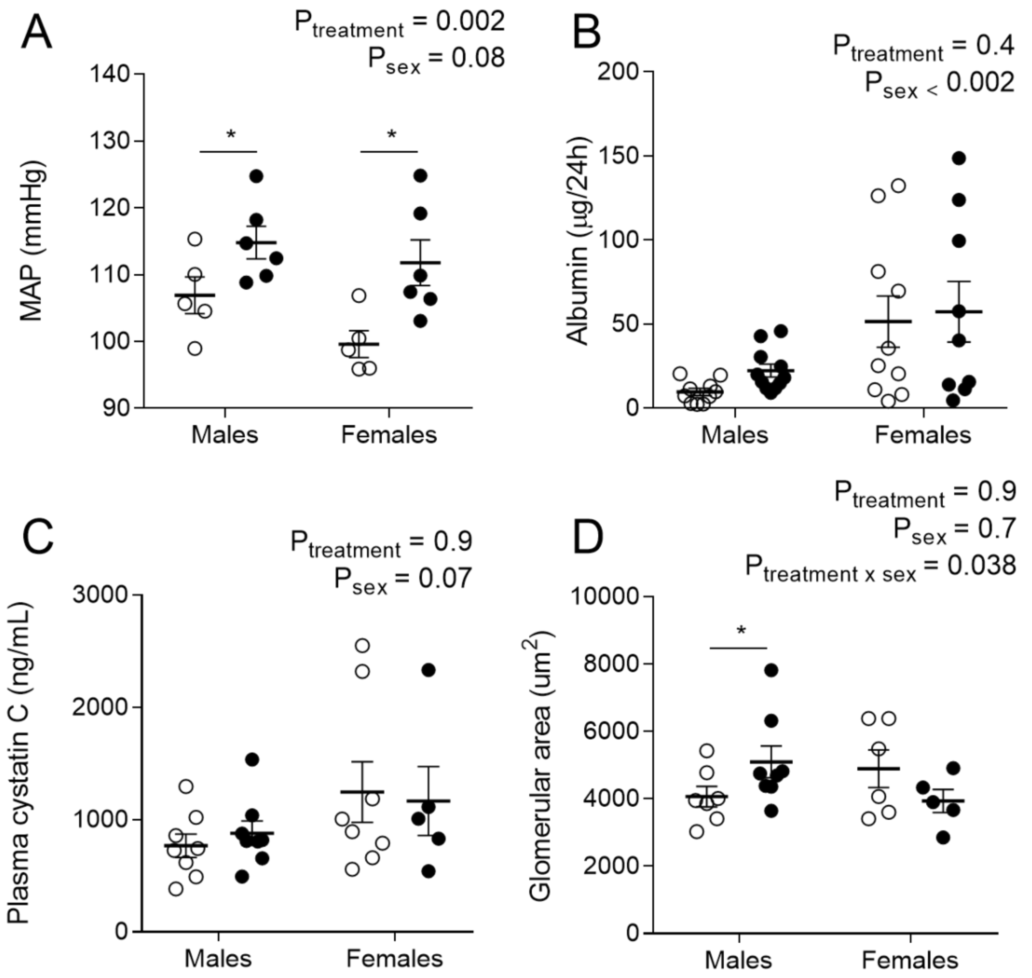

Supplementary Figure S6. Comparison between male and female offspring fed the normal salt diet at 12 months of age.
(A) Mean arterial pressure (MAP, mmHg), (B) albumin excretion (µg/24h), (C) plasma cystatin C (ng/mL) and (D) glomerular area (μm^2). Data were analysed via two-way ANOVA. All data expressed as mean ± SEM. Control: open points; hypoxia: black points. N=5-10 animals per group. *P<0.05 from Sidak *post hoc.*
